# 4-tert-Octylphenol Exposure Disrupts Brain Development and Subsequent Motor, Cognition, Social, and Behavioral Functions

**DOI:** 10.1155/2020/8875604

**Published:** 2020-11-17

**Authors:** Dinh Nam Tran, Eui-Man Jung, Yeong-Min Yoo, Eui-Bae Jeung

**Affiliations:** Laboratory of Veterinary Biochemistry and Molecular Biology, Veterinary Medical Center and College of Veterinary Medicine, Chungbuk National University, Cheongju, Chungbuk 28644, Republic of Korea

## Abstract

The endocrine-disrupting chemical 4-tert-octylphenol (OP) is a widespread estrogenic chemical used in consumer products such as epoxy resins and polycarbonate plastic. However, the effects of OP on brain development are unknown. The present study examined the effects of OP on neuron and neurobehavioral development in mice. By using primary cortical neuron cultures, we found that OP-treated showed a decreased length of axons and dendrites and an increased number of primary and secondary dendrites. OP reduced bromodeoxyuridine (BrdU), mitotic marker Ki67, and phospho-histone H3 (p-Histone-H3), resulting in a reduction of neuronal progenitor proliferation in offspring mouse brain. Moreover, OP induced apoptosis in neuronal progenitor cells in offspring mouse brain. Furthermore, offspring mice from OP-treated dams showed abnormal cognitive, social, and anxiety-like behaviors. Taken together, these results suggest that perinatal exposure to OP disrupts brain development and behavior in mice.

## 1. Introduction

Endocrine-disrupting chemicals (EDCs) have been known as exogenous substances or mixtures that can alter endocrine system functions and consequently cause adverse health effects in humans. Alkylphenols are common EDCs that some industries use for the manufacture of a wide range of products, such as bottles, food packaging, personal care products, and cleaners. Therefore, on a daily basis, humans may be exposed to low doses of alkylphenols, throughout their lives. One of the commonest alkylphenols in consumer products is 4-tert-octylphenol (OP). In Korea, 91.8% of adults aged from 18 to 69 years were observed to have urinary OP concentrations > 0.05ng/mL [1]. Thus, there is increasing concern about the adverse effects of OP exposure on human health. In humans, prenatal OP exposure has been reported to be negatively associated with neonatal size at birth [2]. Moreover, exposure to OP has been associated with idiopathic infertility in Chinese men [3].

Previous studies have often focused on the toxic effects of OP exposure on the reproductive system. There is little information about the effects of OP on brain development and function. In an earlier study, OP concentrations in brain tissue were determined after the termination of OP treatment [4]. Furthermore, OP has been shown to increase the expression of *cyp19a1b*-GFP in the brain of zebrafish embryos [5]. Moreover, OP administered into neonatal female rats may interfere with the sexual differentiation of rat brain [6]. However, reports on the neurotoxic effects of OP are extremely rare.

Additionally, the brain is known as a target organ for the actions of hormones secreted by gonads, adrenals, and the thyroid gland, which begin during the embryonic period with the appearance of hormone receptor sites in neuron [7]. During that period, estrogen has neurotropic, neuromodulating, and neuroprotective effects that influence brain function as well as neuron survival and morphology. Thus, estrogen may influence the memory and cognition mechanisms, as well as motor skills and mood [8]. Moreover, early exposure to estrogenic chemicals as EDCs can alter brain development and function. In humans, the EDC exposure in women has been associated with early neurodevelopmental performance in their offspring at the age of 1–2 years [9]. In mice, prenatal exposure to triclosan impaired brain development and resulting in abnormal behavior [10]. These results suggest that early exposure to EDCs can result in neurodevelopmental disorders and lead to abnormal behavior.

This study is aimed at investigating the effects of OP on neuron and neurobehavioral development. *In vitro*, primary cortical neurons were administered OP over a range of doses (10^−8^ and 10^−6^ M) and showed impaired neuron development. *In vivo*, pregnant mice were subcutaneously treated with OP at doses of 10 mg/kg or 50 mg/kg from gestation day (E) 9.5 to postnatal day (PND) 28. However, the OP treatment impaired neural progenitor proliferation. In addition, behavior assays were conducted in the offspring mice, revealing that offspring from OP-treated dams exhibited social behavior deficits, spatial memory, and learning impairments, as well as cognitive dysfunction, increased anxiety-like behavior, and changed locomotor functions.

## 2. Materials and Methods

### 2.1. Animal Studies

Specific pathogen-free C57BL/6J male and female mice (8 weeks old, 25–30 g) were obtained from Samtaco (Osan, Gyeonggi, Republic of Korea). The mice were housed in polycarbonate cages under controlled environment conditions as in previous [11]. After the acclimatization period, female mice were mated with male mice overnight at a proportion of 2 : 1, and the presence of a vaginal plug was set as embryonic day (E) 0.5. The maternal mice were randomly divided into three groups including vehicle group and two exposure groups (*n* = 5 mouse/group, each mouse was maintained in each cage). Maternal mice were daily received subcutaneous injection of corn oil (vehicle group), or OP (10 mg/kg/day), or OP (50 mg/kg/day) (OP, St. Louis, MO, USA) dissolved in corn oil (Sigma-Aldrich) from E9.5 to postnatal day (PND) 28.5. The 50 mg/kg OP dose was previously reported as the no-observed-adverse-effect level maternal toxicity in rodents [12]. After weaning (PND 28.5), the female and male offspring were separated and housed in group of 3-5 animals.

### 2.2. Primary Cortical Neuron Culture

Primary cortical neuron culture was performed as described [13]. The primary cortical brains were collected from E15.5 mouse embryos and dissociated to single cells after digestion with trypsin (Celgene, Summit, NJ, USA). 1 × 10^5^ neuronal cells were plated in poly-D-lysine coated 24-well plates and cultured in Neurobasal medium/DMEM (1 : 1) with B27 supplement, penicillin, streptomycin, and glutamine (Gibco-BRL, Gaithersburg, MD, USA) at 37°C in a humidified incubator with 5% CO_2_/95% air. The day of plating was considered day *in vitro* 0 (DIV 0). On DIV 1, cells were received OP at a low concentration 10^−6^ M and 10^−8^ M. On DIV 4, neurons were harvested for quantification of axon and dendrite morphology. For proliferative experiments, neurons were incubated with 5-bromo-3-deoxyuridine (BrdU) (10 mM/mL, Sigma-Aldrich, St. Louis, MO, USA) after 12 h treated with OP. Then, neurons were harvest after 2 h BrdU-treatment.

### 2.3. Immunofluorescence

Pregnant females or offspring mice were injected intraperitoneally with BrdU (100 mg/kg) and killed after 2 or 24 h, respectively. Mice were anesthetized with Avertin (2,2,2-tribromoethanol: T48402, Sigma-Aldrich; Ter-amyl alcohol: 240486, Sigma-Aldrich) (0.018 mL (2.5%) per gram of body weight) before they were killed. The brains were collected and briefly fixed in phosphate-buffered 4% paraformaldehyde (PFA) for at 4°C. Then, the brain transfer to 1x PBS and sectioned coronally at 80 *μ*m with a microtome (SM2010R, Leica Microsystems Ltd, Leider Lane, Buffalo Grove, IL, USA). Brain sections were stored in 1x PBS and kept at 4°C until staining.

#### 2.3.1. Staining without BrdU

Neuronal cells or brain sections were fixed in 4% formaldehyde (PFA) and then permeabilized with phosphate buffered saline (PBS) containing Triton X-100 (Sigma-Aldrich) (0.01% for cell). Then, neuron cells or brain sections were blocked in PBS ++ (PBS + 5% goat serum (Vector laboratories, Burlingame, CA, USA) + 0.25% Triton X − 100) for 1 h, followed by incubation in primary antibodies (Cleaved caspase-3, Cell Signaling Technology, Danvers, MA, USA, cat. no. 9661S, 1:400; MAP2, Abcam, Cambridge, UK, cat. no. ab32454, 1 : 500; and Tau1, Abcam, cat. no. ab75714, 1 : 500; Ki67, Cell Signaling Technology, cat. no. D385, 1 : 500; p-Histone-H3, Cell Signaling Technology, cat. no. 9701S, 1 :5 00) at 4°C overnight. For secondary staining, cells were incubated for 1 h in secondary antibody solution (Alexa Fluor488 goat anti-rabbit IgG, cat. no. A11034, 1 : 1000; Alexa Fluor594 goat anti-rabbit, cat. no. A11012, 1 : 1000; and Alexa Fluor488 goat anti-chicken, cat. no. A11039, 1 : 1000, Invitrogen, Carlsbad, CA) that contain 100 ng/mL 4′,6-diamidino-2-phenylindole (DAPI) (Sigma-Aldrich). Then, cells or brain sections were mounted in Fluoro-Gel (Emsdiasum, Hatfield, PA).

#### 2.3.2. BrdU Staining

BrdU staining was performed as described previously [14]. Neuronal cells or brain sections were fixed in 4% PFA for 10 min at room temperature (RT). After permeabilized with PBS containing Triton X-100, neuron cells were incubated in 1 M HCl (Daejung Chemicals & Metals Co., Ltd, Gyeonggi-do, Korea) for 30 min at RT. Then, cells were neutralized in 0.1 M borate buffer (Sigma-Aldrich) for 30 min. Then, cells were blocked PBS ++ for 1 h. For BrdU immunolabeling, cells or brain sections were then incubated in the appropriate primary antibodies BrdU (Bioscience, Durham, NC, USA, cat. no. 555627, 1 : 1000) at 4°C overnight. Then, cells or brain sections were incubated for 1 h in secondary antibody solution (Alexa Fluor594 goat anti-mouse (cat. no. A11032, 1 : 1000, Invitrogen)) that contain DAPI. Following secondary antibody incubations and then were mounted in Fluoro-Gel. Fluorescently labelled cells were visualized using confocal microscopy.

### 2.4. Western Blot Analysis

Brain total protein content was extracted using Pro-prep solution (iNtRON, Seoul, Korea) according to the manufacturer's protocol. 100 *μ*g of protein was resolved by using 12% sodium dodecyl sulfate-polyacrylamide gel electrophoresis and transferred to polyvinylidene fluoride membrane (Merck Millipore, Taunton, MA, USA) as previously described [15]. Then, the membrane was incubated overnight in primary antibodies (Cleaved caspase-3, Cell Signaling Technology, cat. no. 9661S, 1 : 400; Bcl2, Santacruz, cat. no. 7382, 1 : 1000; Bax, Santacruz, cat. no. 7480, 1 : 1000) and secondary antibodies (anti-rabbit, Cell Signaling Technology 1 : 3000; anti-mouse, Cell Signaling Technology 1 : 3000). Membranes were enhanced using chemiluminescence reagent (EMD Millipore Corporation, Burlington, MA, USA). The optical density of the target band was detected with the Chemi Doc equipment, GenGnome5 (Syngene, Cambridge, UK) and analyzed by using the ImageJ software (NIH, Bethesda, MD, USA).

### 2.5. Behavioral Analysis

For experimental design, offspring mice between 6- and 10-week age were randomly selected to perform behavior tests [10, 13].

#### 2.5.1. Open Field Test

The open field test performed in a large acrylic cube measuring by 50 cm tall, 60 cm wide with a white bottom. Briefly, mice were individually placed near the wall side and allowed to freely move for 5 min to measure locomotor activity. The movement of mice was recorded and analyzed with the EthoVision XT14 software (Noldus, Leesburg, VA, USA). Time spent in the center zone (15 × 15cm imaginary square), velocity, and distance travelled was evaluated.

#### 2.5.2. Social Interaction

The subject mouse was introduced in the same open field apparatus with the present of one stranger C57BL/6J mouse and free allowed to explore for 10 min. The interaction indexes including general sniffing, anogenital sniffing, following, mounting, and fighting were counted.

#### 2.5.3. Morris Water Maze

The Morris water maze test was conducted in a circular pool (90 cm diameter, 40 cm high), filled with water (25 ± 1°C), and the water was made opaque by adding skim milk. The tank was divided into four equally sections (I, II, III, IV). A circular platform (10 cm diameter, 20 cm high) made of plexiglass was placed in the middle of a target quadrant (III) (12 cm from the pool's edge and 1 cm below the surface of the water) with visual cues on the pool walls as spatial references. Mice received a two-phase training protocol for 9 days: cue training (4 days) and followed by spatial training (5 days); four trials were performed per mouse per day, and the escape latency (time to find the hidden platform) in each trial was recorded. For each trial, the subject mice were gently placed into the water facing the wall from one of three quadrants (I, II, and IV), varied by day of testing. Then, mice were given 60 s to find platform, and a trial completed when the mice had found the platform. If the mouse failed to find the platform within 60 s period, it was gently guided onto the platform by the experimenter and allowed to rest on it for 30 s. The mean of escape latencies (second) for 4 trials is represented for the learning result for each mouse. On day 10 (probe test day), the platform was removed from the pool, and the mice were left to search for the platform for 60 s. Videos were recorded and analyzed using EthoVision XT14; the time spent in the target platform and the number of crossing the platform were measured to indicate the memory results.

#### 2.5.4. Novel Object Recognition

First, the subject mice were placed into the open field arena in the presence of two identical objects (2cmwidth × 5cmlength × 9cmtall) and freely explored for 10 min. After 6 h, one of the objects was replaced by a novel object (different shape and color compared to old object), and the subject mice were placed inside the field to explore for another 10 min. The duration of time mice spent to interact with the novel and old objects (sniffing or exploring at distance within 2 cm of the object) was recorded. Videos were analyzed using EthoVision XT14.

#### 2.5.5. Tail Suspension Test

Each mouse was suspended on the edge of a shelf, 50 cm above the surface of a table. The subject mice were allowed to move for 6 min, and the behavior was recorded by a camera. Videos were analyzed using EthoVision XT14. The duration of immobility was recorded in the last 5 min.

#### 2.5.6. Forced Swimming Test

Each mouse was gently placed into a glass cylinder (20 cm height, 15 cm in diameter) filled with water (25 ± 2°C) to a depth of 12 cm. All mice were forced to swim for 5 min and then were removed from the water, dried, and returned to their home cages. 24 h later, they were again placed in the cylinder, and after the initial 1 min acclimatization period, the total duration of immobility was measured for 5 min. Videos were analyzed using EthoVision XT14.

#### 2.5.7. Three-Chamber Social Test

The three-chambered social test was performed as follows [16]. The three-chambered apparatus consisted of three Plexiglas chambers; each chamber was 20cmwidth × 40cmlength × 22cmtall, and the dividing walls have small square openings (10 × 5cm) allowing mice free access into each chamber. Both side chambers contained a cylindrical plastic cage (17 cm in height, a bottom diameter of 8 cm with the bars spaced 1 cm apart) in the corner that used to hold the stranger mice. First, the subject mice were allowed to freely explore all three chambers with an empty plastic cage in each side chamber for the 5 min habituation period. For sociability testing, an unfamiliar C57BL/6J mouse (Stranger 1 (S1)) was then introduced in a cylindrical plastic cage in one of the side chambers and an empty cylindrical plastic cage (Empty (E)) on the other side chamber. Then, the subject mouse was placed in the center chamber and allowed to freely explore all three chambers for 10 min. For social novelty test, the empty plastic cage was replaced with a wild-type stimulus mouse (Stranger 2 (S2)), and the subject mouse again freely explored all three chambers for 10 min. All stranger mice were same sex at the same age with the subject mice and previously habituated to the plastic cages. Time spent in proximity, distance travelled, and heat maps were calculated using the automated software EthoVision XT14. The preference index for each animal was calculated as: Preference index = (*S*1 − *E*)/(*S*1 + *E*) or as (*S*2 − *S*1)/(*S*2 + *S*1), where “E”, “S1,” and “S2” are the time spent in close proximity with empty cage and the stranger animals 1 and 2, respectively.

### 2.6. Statistical Analysis

All statistical analyses were performed by applying two-way ANOVA (unpaired Student's *t*-tests for two population comparisons) or one-way ANOVA (Bonferroni's multiple comparison test). Data were randomly collected and analyzed using the GraphPad Prism software (GraphPad Software, La Jolla, CA, USA). The results are presented as means ± SEM, and a *p* < 0.05 was considered statistically significant. The number of mice and statistical detail for all behavioral assays is described in Supplementary Table 1. Each experiment was performed blind and randomized. Animals were assigned randomly to the various experimental groups. The allocation, treatment, and handling of animals were the same across study groups. All treatment group results were compared to the vehicle group and each other.

## 3. Results

### 3.1. OP Impairs Growth and Development of Primary Cortical Neurons

**Figure 1:**
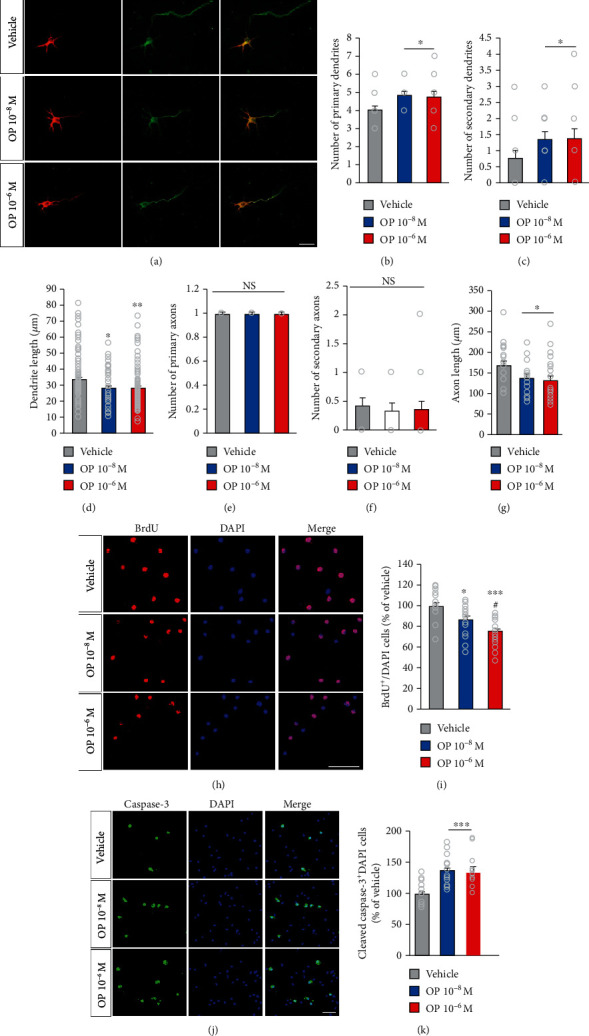
Effect of OP on the growth and proliferation of neuron cell in *in vitro*. (a) Confocal microscope images of cultured mouse primary cortical neuron at DIV 4 stained with antibodies against MAP2 (red) and Tau1 (green). Scale bar: 100 *μ*m. (b–d) Quantification of relative number and length of dendrite. (b) Number of primary dendrites; (c) number of secondary dendrites; (d) average dendrite length. Both the OP 10^−8^ M and OP 10^−6^ M groups showed higher in the number of primary dendrites and secondary dendrites compared to vehicle group. The OP-treated groups displayed a markedly decreased length of primary dendrites compared to the vehicle group. (e–g) Quantification of relative number and length of axon. (e) Number of primary axons; (f) number of secondary axons; (g) average axon length. Both the OP 10^−8^ M and the OP 10^−6^ M groups showed no significant difference in the number of primary axon and secondary axon. The length of axon of primary axons was slightly decreased in the OP-treated groups compared to the vehicle group. *n* = 5 cell culture replicates using 5 mice for each condition (cell counts: 250 cells for each group). (h) Primary cortical neuronal cells were treated OP at DIV 1 and then incubated with 10 *μ*M BrdU for 2 h after 12 h adding OP, fixed and immunofluorescence with BrdU antibodies (red). Scale bar: 40 *μ*m. (i) Quantification of (h). The OP-treated groups exhibited lower in the percentage of cleaved BrdU^+^ cells compared to the vehicle group. The percentage of BrdU^+^ cells in the OP 10^−6^ M group was decreased compared to that in the OP 10^−8^ M group. (j) Cell death was assessed by staining with a cleaved caspase-3 antibody (green) after 12 h adding OP. Scale bar: 100 *μ*m. (k) Quantification of (j). The percentage of cleaved caspase-3^+^ cells was markedly higher in the OP-treated groups compared to the vehicle group. *n* = 5 cell culture replicates using 5 mice for each condition (cell counts: 1,000 cells for each group). Data represent mean ± SEM. Statistical significance was determined by one-way ANOVA with Bonferroni correction. ^∗^*p* < 0.05 vehicle vs. OP, ^∗∗^*p* < 0.01 vehicle vs. OP, ^∗∗∗^*p* < 0.001 vehicle vs. OP. ^#^*p* < 0.05 OP 10^−8^ M vs. OP 10^−6^ M. NS: no significance.

First, to identify the potential alteration neuron, primary cortical neurons were exposed to OP at two low concentrations at day *in vitro* (DIV) 1 and fixed at DIV 4. We traced the morphology of primary cortical neurons in combination with microtubule-associated protein-2 (MAP2) staining and Tau1 to determine average lengths and numbers of axons and dendrites (Figure 1(a)). The numbers of primary and secondary dendrites in the OP-treated groups were markedly higher than those in the vehicle group (Figures 1(b) and 1(c)). However, there was no significant difference in the numbers of both primary and secondary axons between the OP-treated and vehicle groups (Figures 1(e) and 1(f)). In both the OP 10^−8^ M and OP 10^−6^ M groups at DIV 4, there were significant differences in average axon and dendrite lengths compared to those of the vehicle group (Figures 1(d) and 1(g)). These results indicated that OP may regulate the development and growth of neurons during the early stage of differentiation.

Next, we determine the effects of OP on the proliferation and survival of neuronal progenitor cells. Neuronal progenitor cell proliferation was assessed by using bromodeoxyuridine (BrdU) staining on DIV 2 at 1 day after treatment with OP (Figure 1(h)). The percentages of BrdU^+^ cells in both the OP 10^−8^ M and OP 10^−6^ M groups were significantly lower than those in the vehicle group (Figure 1(i)). In addition, the percentage of BrdU^+^ cells in the OP 10^−6^ M group was significantly lower than that in the OP 10^−8^ M group (Figure 1(i)).

Additionally, the apoptotic cell abundance was assessed on DIV 2 by using cleaved caspase-3 staining (Figure 1(j)). The percentages of cleaved caspase-3^+^ cells were markedly higher in both the OP 10^−8^ M and OP 10^−6^ M groups compared to those in the vehicle group (Figure 1(k)). These results suggested that OP may inhibit proliferation and promote apoptosis of neuronal progenitor cells during the early stage of brain development.

### 3.2. OP Impairs Neural Progenitor Cell Premature Differentiation and Maintenance In Vivo

**Figure 2:**
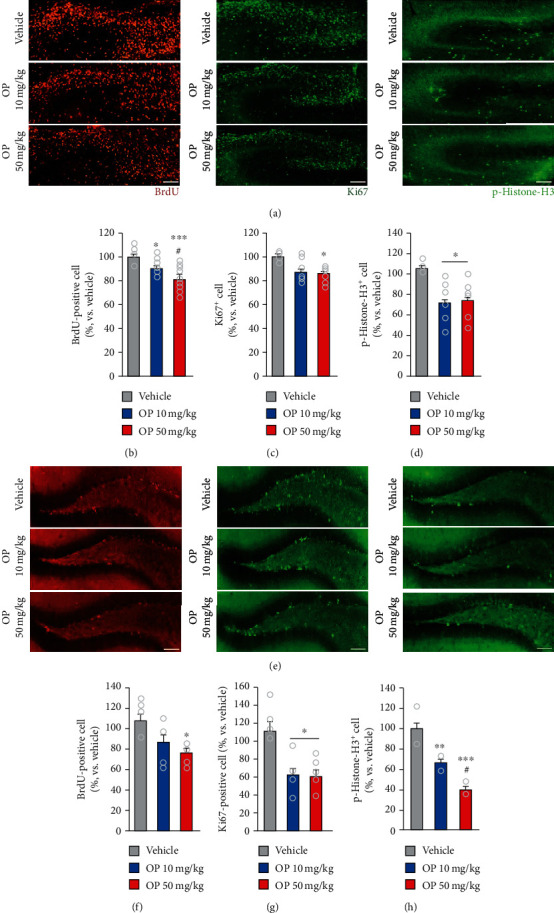
OP exposure alters neurogenesis. (a, e) Nuclei of cells in S phase of cell cycle are labeled using BrdU antibody (red), progenitor cells are labeled using Ki67 (green), and mitotic cells are labeled with phospho-histone H3 antibody (green) on confocal sections of the DG area. The brains were dissected from the vehicle- and OP-treated groups at E18.5 (a) and offspring mice (e). (b–d) Quantification analysis of (a). Histograms show the percent of BrdU^+^, Ki67^+^, and phospho-histone H3^+^ in the DG (*n* = 5 per group). (b) The number of BrdU^+^ cells was decreased in the OP-treated groups compared to the vehicle group at E18.5. The OP 50 mg/kg group showed lower in the number of BrdU^+^ cells compared to the OP 10 mg/kg group (*F*_2,26_ = 4.191, *p* = 0.0001). (c) The number of Ki67^+^ cells was decreased in the OP 50 mg/kg group compared to the vehicle group. There was no difference in the number of Ki67^+^ cells between the OP 10 mg/kg and the vehicle groups (*F*_2,26_ = 4.191, *p* = 0.0302). (d) The OP-treated groups exhibited lower in the number of p-Histone-H3^+^ cells (*F*_2,26_ = 4.191, *p* = 0.0081). (f–h) Quantification analysis of (e). (f) The number of BrdU^+^ cells was decreased in the OP 50 mg/kg group compared to the vehicle group (*n* = 5 per group). The OP 10 mg/kg group shows no significant difference in the number of BrdU^+^ cells compared to the vehicle group (*F*_2,13_ = 4.289, *p* = 0.0371). (g) The OP-treated groups exhibited lower in the number of Ki67^+^ cells (*F*_2,13_ = 6.325, *p* = 0.0121). (h) The OP 50 mg/kg group exhibited markedly lower in the number of p-Histone-H3^+^ cells than the vehicle and OP 10 mg/kg groups. The number of p-Histone-H3^+^ cells was also decreased in the OP 10 mg/kg group compared to the vehicle group (*F*_2,9_ = 34.73, *p* < 0.0001). Data represent mean ± SEM. Statistical significance was determined by one-way ANOVA with Bonferroni correction. ^∗^*p* < 0.05 vs. vehicle, ^∗∗^*p* < 0.01 vs. vehicle, ^∗∗∗^*p* < 0.001 vs. vehicle. ^#^*p* < 0.05 OP 10 mg/kg vs. OP 50 mg/kg. Scale bars: 50 *μ*m in (a); 200 *μ*m in (e).

Next, we examined the effect of perinatal exposure to OP on the proliferation of precursor neuronal cells during embryogenesis. To do so, we determined the number of S-phase cells in the dentate gyrus (DG) area of OP-treated and vehicle embryos at E18.5 by injecting pregnant females with BrdU and harvesting embryos 2 h later, thereby enabling the detection of cells undergoing DNA replication at that time (Figure 2(a)). Immunostaining showed that the number of BrdU^+^ cells was slightly decreased in the E18.5 OP-treated groups compared to that in the vehicle group (Figures 2(a) and 2(b)). Moreover, the number of BrdU^+^ cells in the OP 50 mg/kg group was significantly lower than that in the OP 10 mg/kg group (Figures 2(a) and 2(b)). In addition, the number of cells expressing the mitotic marker Ki67^+^ at DG was markedly lower in the OP 50 mg/kg group than in the vehicle group (Figures 2(a) and 2(c)). However, the number of Ki67^+^ cell in the OP 10 mg/kg group was no significant decreased compared to that in the vehicle and OP 50 mg/kg group (Figures 2(a) and 2(c)). Similarly, the numbers of cells expressing the mitotic marker phospho-histone H3 (p-Histone-H3) at the DG were lower in the OP-treated groups than in the vehicle group at E18.5 (Figures 2(a) and 2(d)). There was no difference in the number of p-Histone-H3^+^ cells between the OP 10 mg/kg and the OP 50 mg/kg groups (Figures 2(a) and 2(d)).

In offspring mice, the number of BrdU^+^ cells in the DG area of the OP 50 mg/kg-treated group was lower than that in the vehicle group (Figures 2(e) and 2(f)). However, there was no difference in the number of BrdU^+^ cells in the DG between the OP 10 mg/kg and the vehicle groups (Figures 2(e) and 2(f)). In addition, the numbers of Ki67^+^ cell in the DG were markedly lower in the OP-treated groups than those in the vehicle group (Figures 2(e) and 2(g)). There was no difference in the number of Ki67^+^ cells in the DG between the OP 10 mg/kg and the OP 50 mg/kg groups. Furthermore, the numbers of p-Histone-H3^+^ cells were significantly lower in the OP-treated groups than in the vehicle group (Figures 2(e) and 2(h)). Also, the number of p-Histone-H3^+^ cells in the OP 50 mg/kg group was lower than that in the OP 10 mg/kg group (Figures 2(e) and 2(h)). These data suggested that perinatal exposure to OP disrupts neurogenesis in the mouse brain.

### 3.3. OP Exposure Affects Progenitors Cycling, Promoting Cell Cycle Exit and Inhibit Cell Cycle Reentry

**Figure 3:**
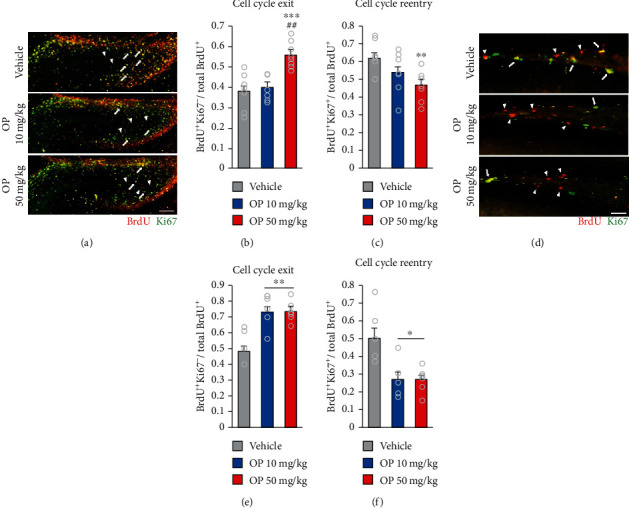
OP exposure impairs the number of cycling in dentate gyrus progenitors by increasing cell cycle exit. (a) Coronal section of E18.5 DG is stained with antibodies against Ki67 (green) and BrdU (red). Arrowheads indicate cell cycle exit cells (BrdU-positive/Ki67-negative). Arrows indicate reentry cell cycle cells (BrdU-positive/Ki67-positive). The cell cycle exit rate was calculated by dividing the number of BrdU-positive/Ki67-negative cells by the total number of BrdU-positive cells in the DG. The cell cycle reentering was calculated by dividing the number of BrdU-positive/Ki67-positive cells by the total number of BrdU-positive cells. (b) Increased proportion of cells exiting the cell cycle in the OP 50 mg/kg group compared to the vehicle and OP 10 mg/kg groups. There was no significant different between the vehicle and the OP 10 mg/kg groups (*F*_2,20_ = 14.93, *p* = 0.0001). (c) The OP 50 mg/kg group exhibited lower number of cells reentering the cell cycle compared to the vehicle group. The number of cells reentering the cell cycle in the OP 10 mg/kg group showed decreased number of cells reentering the cell cycle compared to that in the vehicle group but not significant different (*F*_2,20_ = 5.207, *p* = 0.0151). (d) Coronal images of BrdU/Ki67 colabelling in DG. (e) The OP-treated groups showed increased proportion of cells exiting the cell cycle at the DG compared to the vehicle group (*F*_2,13_ = 7.774, *p* = 0.0054). (f) The number of cells reentering the cell cycle in the OP-treated groups were lower compared to that in the vehicle group. There was no difference in the number of cells exiting rate and cells reentering the cell cycle between the OP 10 mg/kg and the OP 50 mg/kg groups (*F*_2,13_ = 8.089, *p* = 0.0052). *n* = 5 per group. Data represent mean ± SEM. Statistical significance was determined by one-way ANOVA with Bonferroni correction. ^∗^*p* < 0.05 vs. vehicle, ^∗∗^*p* < 0.01 vs. vehicle, ^∗∗∗^*p* < 0.001 vs. vehicle. ^##^*p* < 0.01 OP 10 mg/kg vs. OP 50 mg/kg. Scale bar: 50 *μ*m in (a) and (d).

Data presented suggested perinatal exposure to OP impairs to the neural progenitor cell proliferation and survival, which next lead us to analyses the cell cycle regulation in the OP-treated mice DG. At E18.5, we found that the proportion of cell cycle exit (BrdU^+^Ki67^−^/BrdU^+^) was significantly increased in the OP 50 mg/kg group compared to the vehicle and OP 10 mg/kg groups (Figures 3(a) and 3(b)). In contrast, a significant lower proportion of cells was observed to reentry the cycle in the OP 50 mg/kg group compared to the vehicle group (BrdU^+^Ki67^+^/BrdU^+^) (Figures 3(a) and 3(c)). However, there was no significant difference in the proportion of cell cycle exit and reentry between the vehicle and the OP 10 mg/kg groups (Figures 3(b) and 3(c)). In addition, the OP 50 mg/kg group showed no significant lower in the proportion of cell cycle reentry than the OP 10 mg/kg group (Figures 3(b) and 3(c)).

In offspring DG, the OP-treated groups showed increased proportion of cell cycle exit. However, the OP-treated groups decreased the proportion of cell cycle reentry compared to the vehicle group (Figures 3(d)–3(f)). There was no significant difference in the proportion of cell cycle exit and reentry between the OP 10 mg/kg and the OP 50 mg/kg groups (Figures 3(d) and 3(f)). These may suggest that perinatal exposure to OP affects cell cycle exit and cell cycle reentry of DG neural progenitors in embryonic and adult neurogenesis.

### 3.4. OP Promotes Cell Death in Offspring Mouse Brain

**Figure 4:**
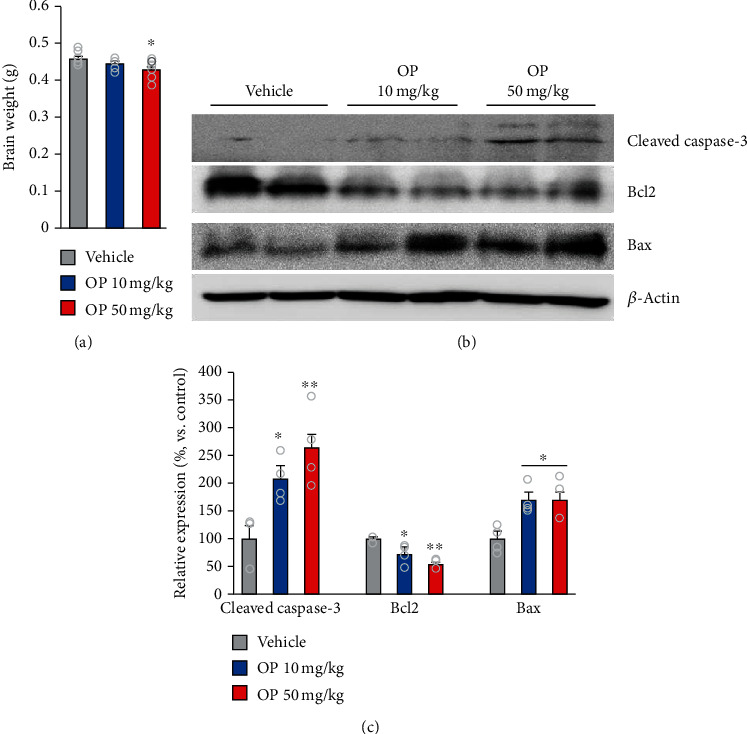
OP promotes cell death in mouse offspring brain. (a) There was significantly lower in the brain weight in the OP 50 mg/kg group compared to vehicle (*F*_2,24_ = 4.508, *p* = 0.0244). (b) Total brain cleaved caspase-3, Bcl2, and Bax protein content were analyzed by using Western blotting (*β*-actin as loading control). (c) Quantification of (b). There were markedly higher in the protein level of cleaved caspase-3 in the OP-treated groups compared to the vehicle group (*F*_2,9_ = 10.84, *p* = 0.0040). In addition, the expressions of Bax protein were also higher in the OP-treated groups compared to the vehicle group (*F*_2,9_ = 7.591, *p* = 0.0117). In contrast, the expressions of Bcl2 protein were significantly lower in the OP-treated groups compared to the vehicle (*F*_2,9_ = 14.21, *p* = 0.0016). *n* = 6 mice (4 males, 2 females) for vehicle, 6 mice (4 males, 2 females) for OP 10 mg/kg, 6 mice (4 males, 2 females) for OP 50 mg/kg. Data represent mean ± SEM. Statistical significance was determined by one-way ANOVA with Bonferroni correction. ^∗^*p* < 0.05 vs. vehicle, ^∗∗^*p* < 0.01 vs. vehicle.

To further elucidate the effects of OP on brain development and neurodevelopment outcomes, maternal mice were subcutaneously treated with OP from E9.5 to PND 28. We observed that the brain weight of offspring mice in the OP 50 mg/kg group was significantly lower (about 7%) than that in the vehicle group (Figure 4(a)). Additionally, the levels of apoptosis signaling in offspring mouse brain in the OP-treated groups were significantly higher than those in the vehicle group (Figures 4(b) and 4(c) and Supplementary Figure 1). Indeed, the OP 10 mg/kg and OP 50 mg/kg groups exhibited a significantly higher level of the protein cleaved caspase-3, a proteolytic product of the apoptotic executioner caspase-3, than that of the vehicle group (Figures 4(b) and 4(c)). In addition, the protein level of antiapoptotic Bcl2 was significantly lower in the OP 10 mg/kg and OP 50 mg/kg groups than in the vehicle group (Figures 4(b) and 4(c)). Moreover, the protein level of proapoptotic Bax was markedly higher in the OP 10 mg/kg and OP 50 mg/kg groups than in the vehicle group (Figures 4(b) and 4(c)). These results indicated that exposure to OP-elevated apoptosis in offspring mouse brain.

### 3.5. OP Exposure Induces Cognitive Dysfunction in Offspring Mice

**Figure 5:**
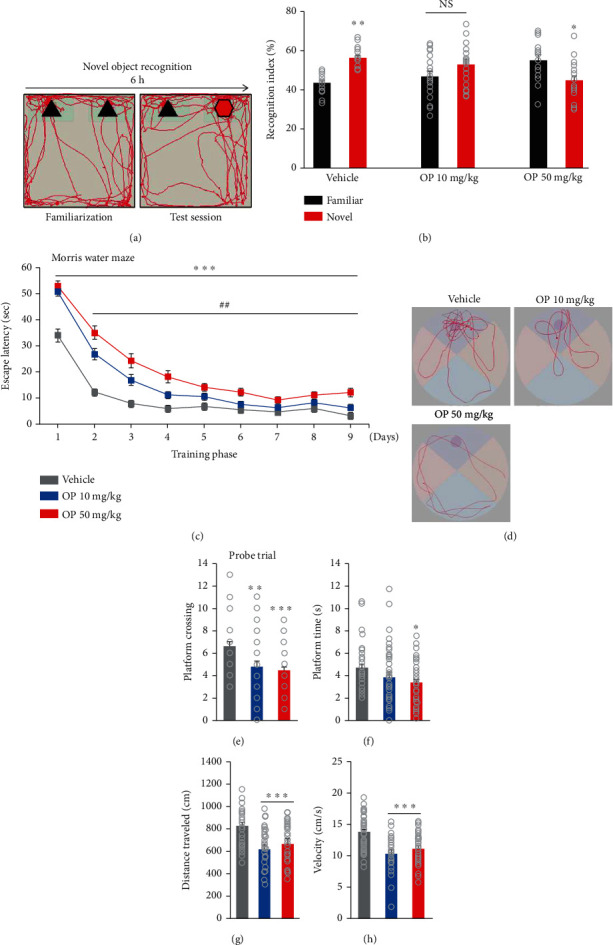
OP impaired learning and recognition memory in mice. (a) Schematic diagram of the novel object recognition test. (b) The vehicle group displayed more time approaching and in proximity to the novel object (vehicle; Familiar = 43.70 ± 1.28, vehicle; Novel = 56.30 ± 1.28, *t* = 6.348, *p* < 0.0001). The OP 10 mg/kg group showed no change in the time spent in novel object compared to vehicle (OP 10 mg/kg; Familiar = 46.98 ± 2.69, OP 10 mg/kg; Novel = 53.02 ± 2.69, (*t* = 1.586, *p* = 0.1219)). OP 50 mg/kg group displayed a significant lower in time spent in novel object (OP 50 mg/kg; Familiar = 55.27 ± 2.72, OP 50 mg/kg; Novel = 45.73 ± 2.72, (*t* = 2.738, *p* = 0.0106)). *n* = 15 mice (8 males, 7 females) for vehicle, 18 mice (8 males, 8 females) for OP 10 mg/kg, 15 mice (8 males, 7 females) for OP 50 mg/kg. Statistical significance was determined by two-way ANOVA, ^∗^*p* < 0.05 familiar vs. novel object. ^∗∗^*p* < 0.01 familiar vs. novel object. NS: no significance. (c) In Morris water maze test, the mean escape latency for trained mice decreased over the course of the 9 learning days in all groups, but the vehicle group significantly improved the latency to locate the platform compared to the OP-treated groups; *n* = 13 mice (7 males, 6 females) for vehicle, 18 mice (9 males, 9 females) for OP 10 mg/kg, 14 mice (9 males, 5 females) for OP 50 mg/kg. Statistical significance was determined by one-way ANOVA. ^##^*p* < 0.01 vehicle vs. OP 10 mg/kg. ^∗∗∗^*p* < 0.001 vehicle vs. OP. ^##^*p* < 0.01 OP 10 mg/kg vs. OP 50 mg/kg. (d) Representative swimming paths of vehicle- and OP-treated mice during a probe trial after training. (e–h) Quantification of (d). The OP-treated groups showed a slightly decrease in platform crossings compared to the vehicle group (*F*_2,128_ = 9.239, *p* = 0.0002). The OP 50 mg/kg group spent less time in platform compared to the vehicle group (*F*_2,124_ = 22.33, *p* = 0.0448). The OP-treated groups changed the swimming distances (*F*_2,132_ = 22.33, *p* < 0.0001) and swimming speeds (*F*_2,132_ = 21.82, *p* < 0.0001). Data represent mean ± SEM. Statistical significance was determined by one-way ANOVA with Bonferroni correction. ^∗^*p* < 0.05 vs. vehicle, ^∗∗^*p* < 0.01 vs. vehicle, ^∗∗∗^*p* < 0.001 vs. vehicle. ^##^*p* < 0.01 OP 10 mg/kg vs. OP 50 mg/kg.

Initially, we assessed whether postpartum female mice exposed to OP showed normal maternal behavior, including pup retrieval, maternal aggression, and milk spots in the stomach of offspring mice. We found that there were no differences between the vehicle- and OP-treated groups that were observed (data not shown). To assess whether prenatal exposure to OP impairs the general behavior of offspring mice, we first assessed recognition memory by using a novel object recognition test (Figures 5(a) and 5(b)). The vehicle group spent more time approaching and in the proximity of the novel object (unfamiliar object) (Figure 5(b)), whereas the OP 10 mg/kg group showed no preference for exploring either familiar or novel objects. Furthermore, the OP 50 mg/kg group had significantly lower times approaching and in proximity to the novel object (Figure 5(b)). Our results suggest that prenatal exposure to OP impairs cognitive functioning in areas such as spatial and nonspatial learning and memory.

Next, we performed the Morris water maze test to evaluate spatial reference learning and memory impairment. All mice were able to learn the platform location during the 4-day acquisition phase (4 trials per day for four successive days) as evidenced by the reduced latency needed to locate the hidden platform. However, both the OP 10 mg/kg and OP 50 mg/kg groups exhibited an increase in the time to find the hidden platform during the acquisition phase (Figure 5(c)). In addition, compared to the OP 10 mg/kg group, the OP 50 mg/kg group required more time to find the hidden platform (Figure 5(c)). On days 5 to 9, the OP 10 mg/kg group reduced the time needed to find the platform, but it was still markedly higher than that of the control mice (Figure 5(c)). The OP 50 mg/kg group exhibited no daily changes in the time to find the platform, suggesting that the capacity for spatial learning in the OP 50 mg/kg group had been limited, rather than delayed (Figure 5(c)). Additionally, there were significantly higher times needed to find the hidden platform in the OP 50 mg/kg group than in the OP 10 mg/kg group (Figure 5(c)). In the probe test, the platform was removed from the pool after completion of the training phase. We observed that both the OP 10 mg/kg and the OP 50 mg/kg groups performed fewer crossings over the former platform site compared to those of the vehicle group (Figure 5(e)). Moreover, compared to the vehicle group, the OP 50 mg/kg group spent less time in the area in which the platform was originally located (Figure 5(f)). Visual representation of the results showed that, compared to the vehicle group, the OP-treated groups had lower proximities to the old platform quadrant (Figure 5(d)). Additionally, the OP-treated groups had markedly decreased swimming distances (Figure 5(g)) and swimming speeds (Figure 5(h)). There was no significant difference between the OP 10 mg/kg and the OP 50 mg/kg groups (Figures 5(e) and 5(h)). Furthermore, there were no significant differences between genders (data not shown). These results indicate that perinatal exposure to OP impairs spatial memory in mice.

### 3.6. OP Induces Social Deficiency in Mice

**Figure 6:**
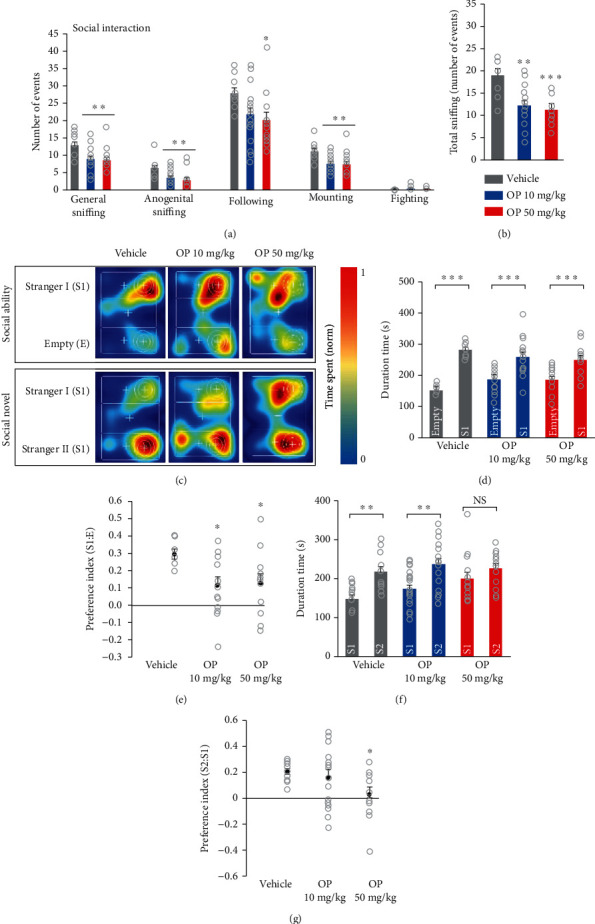
OP mice display social behavioral alteration in three-chamber tests. (a) Social interaction test was performed by using an open field apparatus. The OP-treated groups showed decreased interaction compared to the vehicle group (general sniffing, anogenital sniffing, following). (b) Quantification of total sniffing. The OP-treated groups displayed lower in the total sniffing time compared to the vehicle group (*F*_2,43_ = 9.991, *p* = 0.0003). Statistical significance was determined by one-way ANOVA with Bonferroni correction. ^∗^*p* < 0.05 and ^∗∗^*p* < 0.01 vs. vehicle, ^∗∗∗^*p* < 0.001 vehicle vs. OP. *n* = 13 mice (7 males, 6 females) for vehicle, 18 mice (9 males, 9 females) for OP 10 mg/kg, 15 mice (8 males, 7 females) for OP 50 mg/kg. (c) Representative heat map images of the three-chamber test explaining procedure of sociability and novelty 10 min sessions. (d, e) During sociability session, the vehicle- and OP-treated groups spent more time in the chamber containing an unfamiliar mouse “S1” than in the empty chamber (vehicle; Empty = 152.25 ± 5.17, StrangerI = 282.99 ± 6.57, (*t* = 6.064, *p* < 0.0001); OP 10 mg/kg; Empty = 185.58 ± 8.87, StrangerI = 258.90 ± 14.17, (*t* = 4.385, *p* = 0.0001); OP 50 mg/kg; Empty = 185.25 ± 10.71, StrangerI = 250.75 ± 12.46, (*t* = 4.364, *p* = 0.0002)). However, the OP-treated group preference index was markedly lower compared to the vehicle group (*F*_2,34_ = 5.027, *p* = 0.0122). (f) During social novelty sessions, the vehicle and OP 10 mg/kg groups spent more time in novel chamber “S2” than in familiar chamber “S1”(vehicle; StrangerI = 147.52 ± 7.96, StrangerII = 217.94 ± 12.24, (*t* = 4.061, *p* = 0.0005); OP 10 mg/kg; StrangerI = 172.49 ± 10.63, StrangerII = 237.47 ± 14.01, (*t* = 3.694, *p* = 0.0008)). However, the OP 50 mg/kg group spent almost the same amount of time in the two chambers (OP 50 mg/kg; StrangerI = 200.10 ± 15.61, StrangerII = 227.39 ± 11.36, (*t* = 1.781, *p* = 0.0858)). (g) Social novelty index in the OP 50 mg/kg group was lower than that in the vehicle group (*F*_2,29_ = 3.639, *p* = 0.0389). Data represent mean ± SEM. Statistical significance was determined between times exploring empty chamber and stranger 1 or times exploring stranger 1 and stranger 2 for each group and condition by a two-tailed Student's *t*-test. For preference index, statistical significance was determined by one-way ANOVA with Bonferroni correction. ^∗^*p* < 0.05 and ^∗∗^*p* < 0.01 vs. vehicle, ^#^*p* < 0.05 OP 10 mg/kg vs. OP 50 mg/kg. *n* = 11 mice (6 males, 5 females) for vehicle, 16 mice (8 males, 8 females) for OP 10 mg/kg, 13 mice (7 males, 6 females) for OP 50 mg/kg.

Next, we assessed social interaction and discrimination of social novelty for each mouse test group by using social interaction and three-chamber social tests. In the social interaction test, the OP-treated groups showed lower sniffing time including general sniffing, anogenital sniffing, following, and mounting compared to those of the vehicle group (Figure 6(a)). In addition, compared to the vehicle group, the amount of total sniffing was significantly decreased in the OP-treated groups (Figure 6(b)). These results demonstrate that prenatal exposure to OP induces social behavior deficits in mice. In the three-chamber social test, the mice first explored the three-chamber apparatus in the presence of an unfamiliar mouse located in one of the side chambers (Figure 6(c)). Both the OP 10 mg/kg and the OP 50 mg/kg groups spent significantly more time in the chamber containing an unfamiliar mouse “S1” than in the empty chamber “E” (Figure 6(d)). However, analysis of the preference index for social interaction indicated significant reductions in social preference in both the OP-treated groups compared to that of the vehicle group (Figure 6(e)). In the three-chamber novelty test, both the vehicle and the OP 10 mg/kg groups showed more preference for the novel chamber “S2” than the familiar chamber “S1” (Figures 6(c) and 6(f)). However, the OP 50 mg/kg group exhibited no preference for the stranger mouse “S2” over the familiar mouse “S1” (Figure 6(f)). Furthermore, there was a markedly lower preference index for the novel stimulus in the OP 50 mg/kg group than that in the vehicle group (Figure 6(g)). The preference index for the novel stimulus in the OP 10 mg/kg group was not significantly different from that in the vehicle group (Figure 6(g)). In addition, there was no significant difference both in the preference index for social interaction and novel stimulus between OP the 10 mg/kg and the OP 50 mg/kg groups (Figures 6(e) and 6(g)). These results demonstrated that prenatal exposure to OP impairs sociability and social novelty preference.

### 3.7. OP Exposure Induces Anxiety-Like Behavior

**Figure 7:**
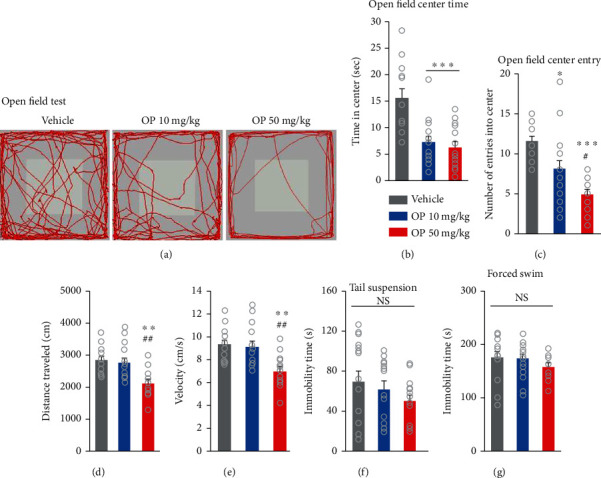
OP impairs locomotor activity, anxiety-like behavior, and nesting behavior in mice. (a) Representative tracing of mouse travel in the open field test. (b–e) Quantification of (a). The OP-treated groups displayed markedly reduction in both open field center time (*F*_2,37_ = 11.69, *p* = 0.0001) and open field center entry compared to the vehicle group (*F*_2,39_ = 14.54, *p* < 0.0001). The OP 50 mg/kg group showed fewer time entry to center compared to the OP 10 mg/kg group. In addition, the OP 50 mg/kg group exhibited significant lower in distance traveled (*F*_2,38_ = 9.004, *p* = 0.0006) and velocity in open field compared to both the vehicle group (*F*_2,38_ = 8.996, *p* = 0.0006). *n* = 18 mice (9 males, 9 females) for vehicle, 18 mice (9 males, 9 females) for OP 10 mg/kg, 15 mice (8 males, 7 females) for OP 50 mg/kg. Statistical significance was determined by one-way ANOVA with Bonferroni correction. ^∗^*p* < 0.05 and ^∗∗^*p* < 0.01 vs. vehicle, ^∗∗∗^*p* < 0.001 vs. vehicle, ^#^*p* < 0.05 OP 10 mg/kg vs. OP 50 mg/kg, ^##^*p* < 0.01 OP 10 mg/kg vs. OP 50 mg/kg. (f) In tail suspension test, the OP-treated groups display no significant change in the immobility times compared to the vehicle group (*F*_2,38_ = 1.122, *p* = 0.3361). (g) In forced swimming test, there was no difference in the immobility times between vehicle- and OP-treated groups (*F*_2,41_ = 0.9785, *p* = 0.3845). *n* = 14 mice (7 males, 7 females) for vehicle, 15 mice (8 males, 7 females) for OP 10 mg/kg, 12 mice (7 males, 5 females) for OP 50 mg/kg. Data represent mean ± SEM. Statistical significance was determined by one-way ANOVA with Bonferroni correction. NS: no significance.

We then performed open field testing to analyze motor activity (reflected by the distance traveled) and anxiety-like behavior (reflected by the amount of time in the center zone). Compared to the vehicle group, the OP-treated groups spent significantly reduced time in the open field center (Figures 7(a) and 7(b)). However, there was no significant different in the time in the open field center between the OP 10 mg/kg and the OP 50 mg/kg groups (Figure 7(b)). The OP-treated groups also showed significantly fewer open field center entries compared to those of the vehicle group (Figure 7(c)). Moreover, the OP 50 mg/kg group displayed significantly lower open field center entries than that of the OP 10 mg/kg group (Figure 7(c)). Furthermore, the OP 50 mg/kg group showed markedly lower velocity and less distance traveled compared to both the vehicle mice and the OP 10 mg/kg group (Figures 7(d) and 7(e)). There were no significant differences in the velocity or distance traveled between the OP 10 mg/kg group and the vehicle group (Figures 7(d) and 7(e)). The results suggest that prenatal exposure to a high dose of OP (50 mg/kg) elevates anxiety-like behavior and impairs locomotor activity in mice.

By assessing depressive-like behavior by using tail suspension and forced swimming tests, we examined the possibility that OP could induce depression in mice. The tail suspension test results indicated that the immobility times of the OP-treated groups were not significantly different from that of the vehicle group (Figure 7(f)). There was also no significant difference between the immobility times of the OP 10 mg/kg and OP 50 mg/kg groups (Figure 7(f)). In the forced swimming test, there were no significant differences between the results for the OP-treated groups and those of the vehicle group (Figure 7(g)). These results show that perinatal exposure to OP does not affect the exhibition of depression-like behavior in offspring mice.

## 4. Discussion

The present study has demonstrated that perinatal OP exposure alters neuronal progenitor proliferation and maintains neural progenitors in the brain development. We also observed cell death and smaller brains in OP-treated mice. Moreover, offspring mice derived from dams treated with OP exhibited abnormal behaviors including cognitive dysfunction, deficits in social behavior, and increases in anxiety-like behaviors.

Environmental factors during early brain developmental periods have long-term consequences on brain function in the later time-life [17], and fetal exposure to EDCs has been associated with adverse neurobehavioral outcomes across the lifespan [18]. Perinatal exposure to bisphenol A (BPA) has effects on brain cells [19], increases anxiety-like behavior, and impairs learning and memory ability in offspring mice [20]. Maternal exposure to di-(2-ethylhexyl) phthalate (DEHP) induced abnormal neuronal development and led to impairment of social interaction ability [21]. Moreover, rat administered butylparaben during the period from early pregnancy to weaning produce offspring with autism-like symptoms [22]. Perinatal exposure to triclosan induced neurodevelopment disorder and led to abnormal social behaviors, cognitive impairment, and deficits in spatial learning and memory [10]. These observations suggest that OP may also impair central nervous system development.

Previous studies have reported that EDCs can induce apoptosis and neurotoxicity in mouse cortical cells [23]. Moreover, perinatal exposure to di-(2-ethylhexyl) phthalate impairs the dendritic growth of pyramidal neurons in rat offspring [24]. In addition, PCB exposure produces abnormal dendritic growth of hippocampal neurons [25]. Similarly, in the present study, OP reduced the average length of both axons and dendrites on DIV 4 of primary cortical neuron culture. Moreover, the numbers of primary and secondary dendrites were increased by OP. We also observed that exposure to OP resulted in decreased proliferation of neuronal progenitor cells. In contrast, OP enhanced apoptosis in neuronal progenitor cells. In an earlier study, estrogen was shown to be involved in the neuronal differentiation, cell migration, cell survival, and death of neurons [26]. Furthermore, prolonged OP exposure from E9.5 to E17.5 decreased neural progenitor cell proliferation. Moreover, we determined that perinatal exposure to OP could disrupt neurogenesis in mice, resulting in reduced numbers of BrdU^+^ neuronal precursors, an important observation as all neuron and glial cells are derived from embryonic and postnatal neural stem cells. In addition, the loss of general proliferation rates was found in the perinatal exposure to OP, when using both BrdU and Ki67 proliferative markers. This increased cell cycle exit and decreased the cell cycle reentry in E18.5 brain could lead to the decreased in the number of dividing progenitors and, consequently, of newborn mature cells. In addition, alterations in axon or dendrite morphogenesis have been associated with various neurological and neuropsychiatric disorders such as autism spectrum disorders, Alzheimer's disease, schizophrenia, Down syndrome, Fragile X syndrome, Rett syndrome, anxiety, and depression [27]. These observations indicate that OP can influence neurogenesis and gliogenesis during embryogenesis and can impair the migration and connection of neurons in mice.

Additionally, the brain weights of offspring mice were lower following high dose OP exposure, indicating that exposure to OP at an early gestation stage can impair brain development. The observed decrease in brain weight could be associated with the OP impairment of the proliferation and apoptosis of neural progenitor cells. In present study, OP-treated mice exhibited suppressed progenitor cell proliferation in the DG. Moreover, we also observed that exposure to OP at an early stage of gestation can influence the expression of many well-known apoptosis genes. In particular, the expression of cleaved caspase-3 in offspring mouse brain was upregulated by OP exposure. In addition, OP changed the expression of Bcl2 family proteins in offspring mouse brain. Also, OP promoted the expression of a proapoptotic Bax protein, while decreasing the expression of an antiapoptotic Bcl2 protein in offspring mouse brain. A previous study showed that Bcl2 family proteins activated caspase through intrinsic and extrinsic apoptotic pathways in a model of neuronal apoptosis [28]. Moreover, both antiapoptotic Bcl2 and proapoptotic Bax proteins have been implicated in excitotoxicity, oxidative stress, and trophic factor deprivation-induced neuronal apoptosis [29, 30]. These findings suggest that perinatal exposure to OP promotes apoptosis in the adult brain by regulating the expression level of Bcl2 and Bax. Thus, we conclude that OP impairs the initial production of neurons at the expense of progenitor cells, leading to a decrease of the latter throughout the embryonic nervous system. In addition, OP exposure impaired axon and dendrite development, which is associated with the formation of connections within the brain, causing a decrease in the number of synapses. Moreover, a reduction in synapses appropriately reflects perturbed brain circuitry. These effects may also be related to the lower brain volume and the abnormal behaviors in offspring mice.

Recent studies have shown that perinatal exposure to EDCs is linked to many neuropsychiatric disorders. In the present study, we determined that perinatal exposure to OP could result in impaired learning and memory at both low and high dose levels. Also, offspring mice in the OP high dose group exhibited learning and memory limitations, indicating that OP effects on neurodevelopment could be dosage dependent. In addition, OP was shown to impair cognition, sociability, and social novelty in offspring mice. Moreover, perinatal exposure to OP increased anxiety-like behavior and changed locomotor functions. These abnormal behaviors may be the result of the adverse effects of OP on neurodevelopment during the early gestational stage. Furthermore, the abnormal behaviors induced by maternal exposure to BPA may be involved in synaptic plasticity alteration [31, 32]. Moreover, early life exposure to BPA altered neurotransmitter systems [33]. Perinatal BPA exposure disrupts the dopaminergic and glutamatergic systems [34, 35]. In addition, perinatal BPA exposure decreased in the global level 5-mC DNA and increased in H3 acetylation by modulating the expression of DNA methyltransferase 1 (DNMT1), DNMT3a, and histone deacetylase 2 [36]. Moreover, developmental BPA exposure resulted in the disruption of epigenetic programming of gene expression [37]. These may suggest that perinatal exposure to EDCs induces epigenetic changes that possibly underlie the enduring effect of EDCs on brain functions and behaviors.

In summary, we first observed pronounced neurogenesis defects and behavioral abnormalities in mice offspring of OP-exposed dams. Perinatal exposure to OP not only produced impairment of neurogenesis and neurite growth but also impaired memory and social behavior and increased anxiety behaviors in offspring mice. Therefore, the findings of the present study into OP exposure at early gestational age should encourage thorough consideration of the adverse effects of EDC exposure in humans. Future work will focus on the possible epigenetic actions of OP and how these may relate to OP estrogenic action at the molecular level.

## Data Availability

The data used to support the findings of this study are available from the corresponding author upon request.

## Supplementary Materials

Supplementary materials The supplementary file includes the graphical abstract. Supplementary figure 1: full length Western blots. Supplementary Table 1: behavior statistic data.
