# Supplementary material for: 4-tert-Octylphenol Exposure Disrupts Brain Development and Subsequent Motor, Cognition, Social, and Behavioral Functions
Source: Oxid Med Cell Longev. 2020 Nov 17;2020:8875604. doi: 10.1155/2020/8875604 (PMC7691001; doi:10.1155/2020/8875604)
Supplement: Supplementary materials — The supplementary file includes the graphical abstract. Supplementary figure 1: full length Western blots. Supplementary Table 1: behavior statistic data. [file 8875604.f1.docx]

**Supplementary materials**

**4-tert-octylphenol exposure disrupts brain development and subsequent motor, cognition, social and behavioral functions**

Dinh Nam Tran, Eui-Man Jung, Yeong-Min Yoo, and Eui-Bae Jeung^*^.

**Graphical abstract:**


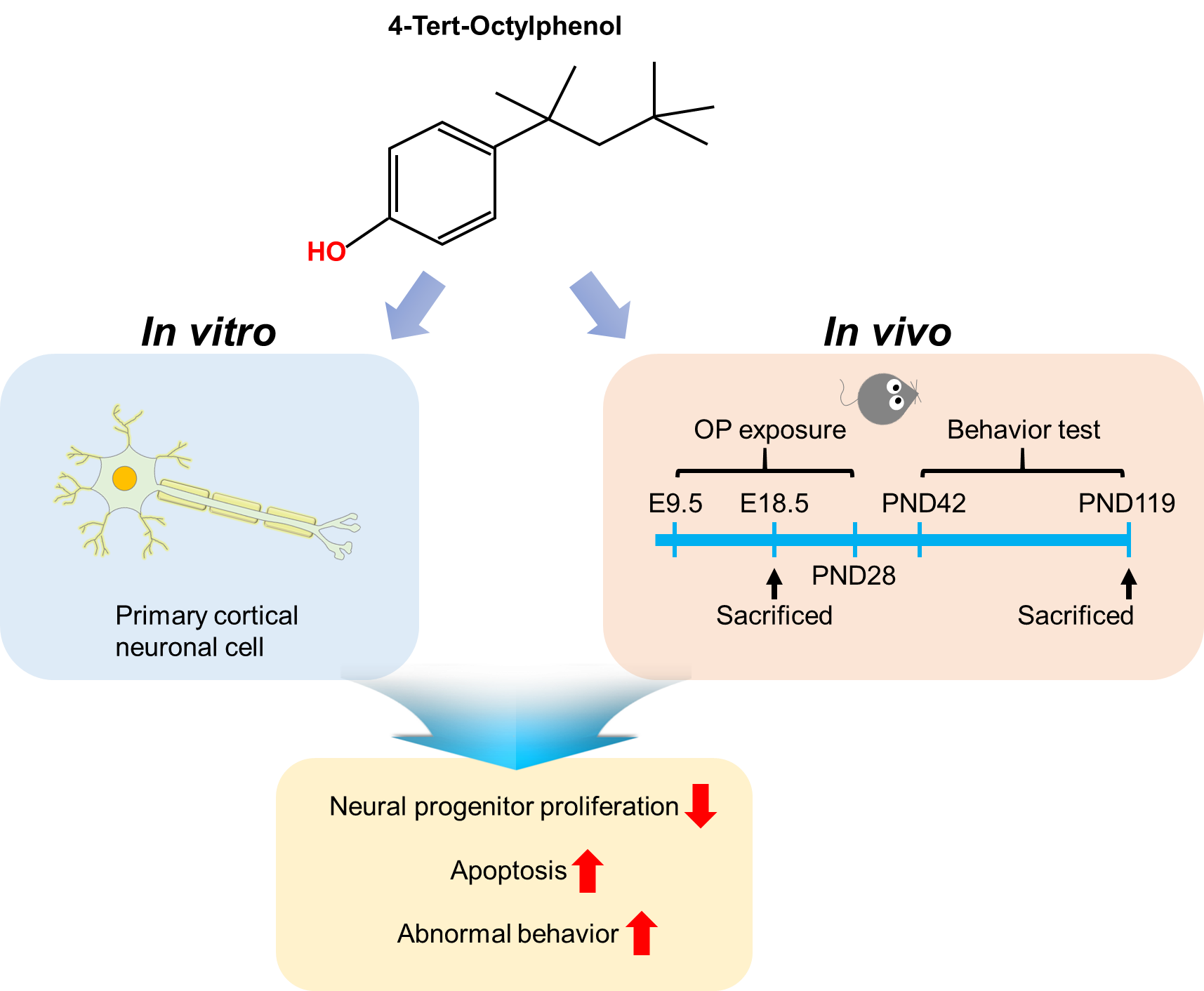


**Supplementary figure 1. Full length Western blots.**

Full length Western blots for cropped images in Figure 4b.


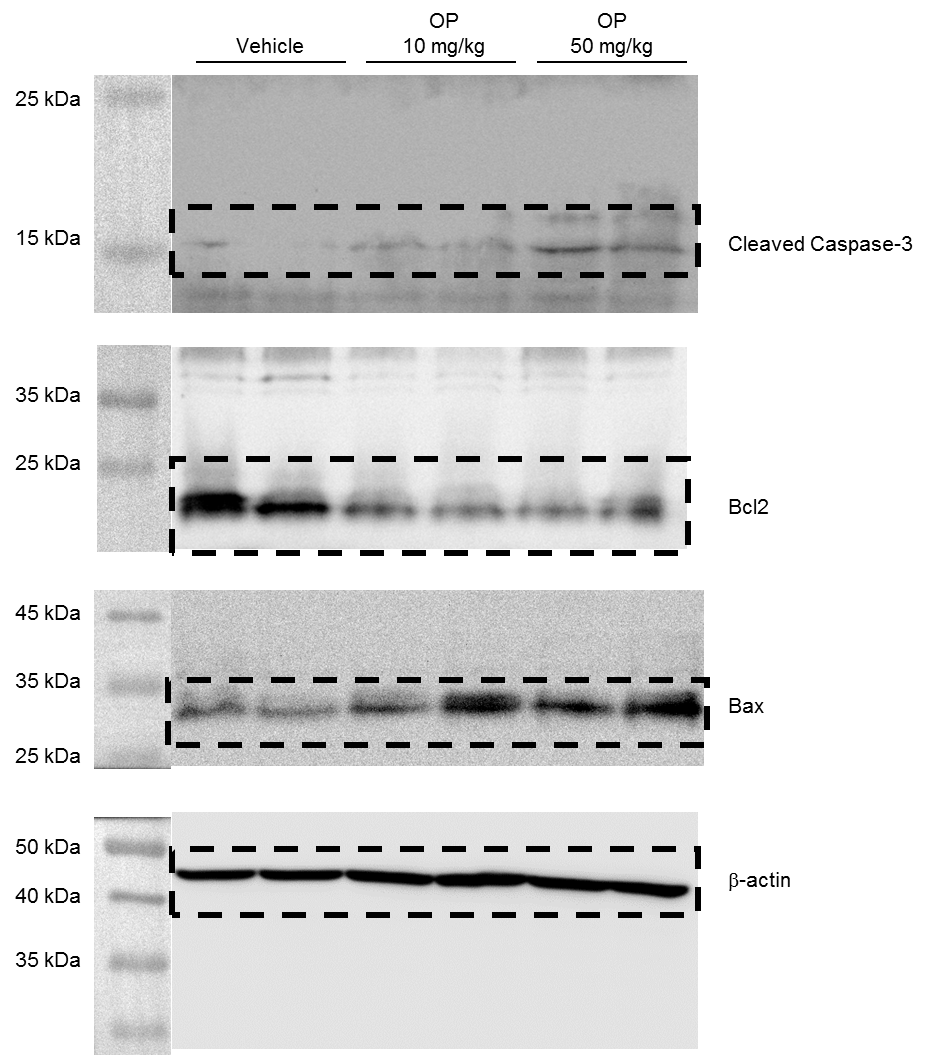


**Supplementary Table 1**. Behavior statistic data

| **Test** | **Duration** | **Measurement** | **Number of animals** | **Values** | | **Statistical test** |
| --- | --- | --- | --- | --- | --- | --- |
|  |  |  |  | Day1 | *VE* = 33.89 ± 2.59 | One-way ANOVA with Bonferroni correction test |
|  |  |  |  |  | *OP10* = 50.85 ± 1.84 |  |
|  |  |  |  |  | *OP50* = 58.98 ±1.81 |  |
|  |  |  |  | Day2 | *VE* = 12.13 ± 0.96 |  |
|  |  |  |  |  | *OP10* = 26.76 ± 2.10 |  |
|  |  |  |  |  | *OP50* = 34.95 ± 2.60 |  |
|  |  |  |  | Day3 | *VE* = 7.68 ± 0.47 |  |
|  |  |  |  |  | *OP10* = 16.89 ± 2.11 |  |
|  |  |  |  |  | *OP50* = 24.23 ± 2.70 |  |
|  |  |  |  | Day4 | *VE* = 5.85 ± 0.44 |  |
|  |  |  |  |  | *OP10* = 11.22 ± 0.75 |  |
|  |  |  |  |  | *OP50* = 18.10 ± 2.25 |  |
|  |  |  |  | Day5 | *VE* = 6.76 ± 0.31 |  |
|  |  |  |  |  | *OP10* = 10.53 ± 0.33 |  |
|  |  |  |  |  | *OP50* = 14.15 ± 1.48 |  |
|  |  |  |  | Day6 | *VE* = 5.35 ± 0.33 |  |
| Morris water maze | 1 min | Escape latency (sec) | VE = 13  OP10 = 18  OP50 = 14 |  | *OP10* = 7.54 ± 0.28 |  |
|  |  |  |  |  | *OP50* = 12.23 ± 1.36 |  |
|  |  |  |  | Day7 | *VE* = 4.54 ± 0.24 |  |
|  |  |  |  |  | *OP10* = 6.39 ± 0.36 |  |
|  |  |  |  |  | *OP50* = 9.13 ± 0.63 |  |
|  |  |  |  | Day8 | *VE* = 5.80 ± 0.43 |  |
|  |  |  |  |  | *OP10* = 8.14 ± 0.60 |  |
|  |  |  |  |  | *OP50* = 11.10 ± 0.64 |  |
|  |  |  |  | Day9 | *VE* = 3.30 ± 0.22 |  |
|  |  |  |  |  | *OP10* = 6.22 ± 0.26 |  |
|  |  |  |  |  | *OP50* = 12.15 ± 1.59 |  |
|  |  | Platform crossing |  |  | *VE* = 6.92 ± 0.40 |  |
|  |  |  |  |  | *OP10* = 4.82 ± 0.47 |  |
|  |  |  |  |  | *OP50* = 4.48 ± 0.28 |  |
|  |  | Distance move (cm) |  |  | *VE* = 8.15 ± 22.76 |  |
|  |  |  |  |  | *OP10* = 621.09 ± 26.66 |  |
|  |  |  |  |  | *OP50* = 670.74 ± 20.21 |  |
|  |  | Velocity (cm/s) |  |  | *VE* = 13.58 ± 0.38 |  |
|  |  |  |  |  | *OP10* = 10.37 ± 0.44 |  |
|  |  |  |  |  | *OP50* = 11.17 ± 0.34 |  |
| Novel object recognition | 10 min | Recognition index (%) | VE = 15  OP10 = 18  OP50 = 15 |  | *VE;* Familiar = 43.70 ± 1.28 | Two-tailed Student’s *t* test |
|  |  |  |  |  | *VE;* Novel = 56.30 ± 1.28 |  |
|  |  |  |  |  | *OP10;* Familiar = 46.98 ± 2.69 |  |
|  |  |  |  |  | *OP10;* Novel = 53.02 ± 2.69 |  |
|  |  |  |  |  | *OP50;* Familiar = 55.28 ± 2.72 |  |
|  |  |  |  |  | *OP50;* Novel = 44.72 ± 2.72 |  |
| Social behavior | 10 min | Time spent in chamber (sec) | VE = 11  OP10 = 16  OP50 = 13 | Sociability | *VE*; Empty = 151.12 ± 5.59 | Two-tailed Student’s *t* test |
|  |  |  |  |  | *VE*; Stranger I = 262.34 ± 12.12 |  |
|  |  |  |  |  | *OP10*; Empty = 185.58 ± 8.87 |  |
|  |  |  |  |  | *OP10*; Stranger I = 258.90 ± 14.17 |  |
|  |  |  |  |  | *OP50*; Empty = 185.25 ± 10.71 |  |
|  |  |  |  |  | *OP50*; Stranger I = 250.75 ± 12.46 |  |
|  |  |  |  | Social novelty | *VE*; Stranger I = 140.11 ± 10.29 |  |
|  |  |  |  |  | *VE*; Stranger II = 222.41 ± 13.81 |  |
|  |  |  |  |  | *OP10*; Stranger I = 172.49 ± 10.63 |  |
|  |  |  |  |  | *OP10*; Stranger II = 237.47 ± 14.01 |  |
|  |  |  |  |  | *OP50*; Stranger I = 195.78 ± 15.66 |  |
|  |  |  |  |  | *OP50*; Stranger II = 230.10 ± 11.50 |  |
| Social interaction | 10 min | Number of events (n) | VE = 13  OP10 = 18  OP50 = 15 | General sniffing | *VE* = 12.08 ± 0.90  *OP10* = 8.83 ± 0.81  *OP50* = 8.53 ± 0.81 | One-way ANOVA with Bonferroni correction test |
|  |  |  |  | Anogenital sniffing | *VE* = 6.23 ± 0.65  *OP10* = 3.39 ± 0.51  *OP50* = 2.8 ± 0.64 |  |
|  |  |  |  | Following | *VE* = 28 ± 1.35  *OP10* = 21.78 ± 1.88  *OP50* = 20.26 ± 2.00 |  |
| Open field | 5 min | Time in center (sec) | VE = 18  OP10 = 18  OP50 = 15 |  | *VE* = 17.05 ± 2.02 | One-way ANOVA with Bonferroni correction test |
|  |  |  |  |  | *OP10* = 7.03 ± 1.08 |  |
|  |  |  |  |  | *OP50* = 6.28 ± 1.03 |  |
|  |  | Number of entries into center |  |  | *VE* = 11.08 ± 0.75 |  |
|  |  |  |  |  | *OP10* = 8.07 ± 1.08 |  |
|  |  |  |  |  | *OP50* = 4.93 ± 0.60 |  |
| Forced swim | 5 min | Immobility time (sec) | VE = 14  OP10 = 15 OP50 = 12 |  | *VE* = 177.09 ± 9.76  *OP10* = 174.18 ± 7.40  *OP50* = 142.78 ± 9.63 | One-way ANOVA with Bonferroni correction test |
| Tail suspension | 5 min | Immobility time (sec) | VE = 14  OP10 = 15  OP50 = 12 |  | *VE* = 69.81 ± 10.27  *OP10* = 68.83 ± 8.81  *OP50* = 41.56 ± 7.44 | One-way ANOVA with Bonferroni correction test |

VE: Vehicle; OP10: OP 10 mg/kg/day; OP50: OP 50 mg/kg/day
